# Predictive performances of animal models using different multibreed relationship matrices in systems with rotational crossbreeding

**DOI:** 10.1186/s12711-022-00714-w

**Published:** 2022-04-06

**Authors:** Bjarke Grove Poulsen, Tage Ostersen, Bjarne Nielsen, Ole Fredslund Christensen

**Affiliations:** 1 Breeding & Genetics, Danish Agriculture and Food Council, Axelborg, Axeltorv 3, Copenhagen W, 1609 Copenhagen, Denmark; 2 Center for Quantitative Genetics and Genomics, Aarhus University, Blichers Allé 20, 8830 Tjele, Denmark

## Abstract

**Background:**

In livestock breeding, selection for some traits can be improved with direct selection for crossbred performance. However, genetic analyses with phenotypes from crossbred animals require methods for multibreed relationship matrices; especially when some animals are rotationally crossbred. Multiple methods for multibreed relationship matrices exist, but there is a lack of knowledge on how these methods compare for prediction of breeding values with phenotypes from rotationally crossbred animals. Therefore, the objective of this study was to compare models that use different multibreed relationship matrices in terms of ability to predict accurate and unbiased breeding values with phenotypes from two-way rotationally crossbred animals.

**Methods:**

We compared four methods for multibreed relationship matrices: numerator relationship matrices (NRM), García-Cortés and Toro’s partial relationship matrices (GT), Strandén and Mäntysaari’s approximation to the GT method (SM), and one NRM with metafounders (MF). The methods were compared using simulated data. We simulated two phenotypes; one with and one without dominance effects. Only crossbred animals were phenotyped and only purebred animals were genotyped.

**Results:**

The MF and GT methods were the most accurate and least biased methods for prediction of breeding values in rotationally crossbred animals. Without genomic information, all methods were almost equally accurate for prediction of breeding values in purebred animals; however, with genomic information, the MF and GT methods were the most accurate. The GT, MF, and SM methods were the least biased methods for prediction of breeding values in purebred animals.

**Conclusions:**

For prediction of breeding values with phenotypes from rotationally crossbred animals, models using the MF method or the GT method were generally more accurate and less biased than models using the SM method or the NRM method.

## Background

Several livestock production systems use crossbred animals at the commercial level. In these systems, the phenotypic performance of crossbred animals should be the primary objective of the breeding goal.

Crossbred performance is often indirectly selected for through selection for purebred performance. This is valid if the genetic correlation between the crossbred and purebred performances is strong [1]. However, the genetic correlation between crossbred and purebred performances is only moderate for many traits [2]. For such traits, it may be a solution to directly select for crossbred performance.

Multiple crossbreeding procedures exist [3]. The most notable procedures for modern pork and beef systems are the two-way terminal, three-way terminal, and two-way rotational crossbreeding procedures. Among these crossbreeding systems, genetic analysis is most complicated with phenotypes from rotationally crossbred animals [4–6]. Nevertheless, rotationally crossbred animals comprise a possible source of both additional and novel phenotypes. Therefore, in the following, we will focus on genetic analyses with phenotypes from rotationally crossbred animals.

Phenotypes from rotationally crossbred animals are often subject to more variable genetic effects than phenotypes from purebred animals and F1 animals [4, 7]. Mating animals from different populations often leads to offspring with a high degree of heterozygosity. For dominance effects, the increase in heterozygosity results in a favorable change in the phenotypic mean and increased dominance variance in subsequent generations [7]. For additive genetic effects, the increase in heterozygosity increases the additive genetic variance in following generations [4]. All the aforementioned changes are relative to the average of the genetic parameters in the constituting purebred populations. Animal breeding focuses mainly on additive genetic effects, which are modelled using additive genetic relationship matrices. Since the usual numerator relationship matrix (NRM) [8] can not correctly model additive genetic effects in rotationally crossbred animals [4], specialized additive relationship matrices are needed.

Specialized additive genetic relationship matrices for crossbred animals exist [4, 9–11]. These relationship matrices decompose the additive genetic relationships into a breed-specific term for each breed and a segregation term for each pair of breeds. The partial relationship matrices for the breed-specific terms are analogous to NRM-based matrices and they refer to the additive genetic variances in the purebred base populations. Meanwhile, the partial relationship matrices for the segregation terms model the increased additive genetic variances in crossbred animals. Both types of partial relationship matrices have later been approximated [12] and the theory for the partial relationship matrices for breed-specific terms has been extended to incorporate genomic information [13]. The additive relationship matrix with metafounders was proposed by Legarra et al. [14], and it is an alternative to the partial relationship matrices mentioned above. In theory, the relationship matrix with metafounders simultaneously models both breed-specific and segregation terms with one additive genetic effect [14]. There is a need to investigate how models with these relationship matrices compare for prediction of accurate and unbiased breeding values with phenotypes from rotationally crossbred animals.

The objective of this study was to compare methods for relationship matrices in terms of ability to predict accurate and unbiased breeding values with phenotypes from rotationally crossbred animals. We compared the NRM as used by Poulsen et al. [15], the partial relationship matrices by García-Cortés and Toro [9], the approximate partial relationship matrices by Strandén and Mäntysaari [12], and the relationship matrix with metafounders by Legarra et al. [14].

We hypothesized that the methods by García-Cortés and Toro [9] and Legarra et al. [14] were the most accurate and least biased methods because they are the only methods which fully comply with the theory [4].

## Methods

The prediction accuracies and prediction biases of the models with different relationship matrices were investigated through a simulation study. The simulation design represents a two-way-rotational crossbreeding system [3]. In this section, we first present how the populations were simulated. This includes the description of their population structure, genomic architecture, genetic effects, and phenotypes. Then, we present how we predicted breeding values with phenotypes from rotationally crossbred animals using statistical models with different relationship matrices. Lastly, we present how we evaluated and compared the statistical models with different relationship matrices. In the following, we refer to the statistical models with different relationship matrices as methods.

### Simulation

#### General

**Fig. 1:**
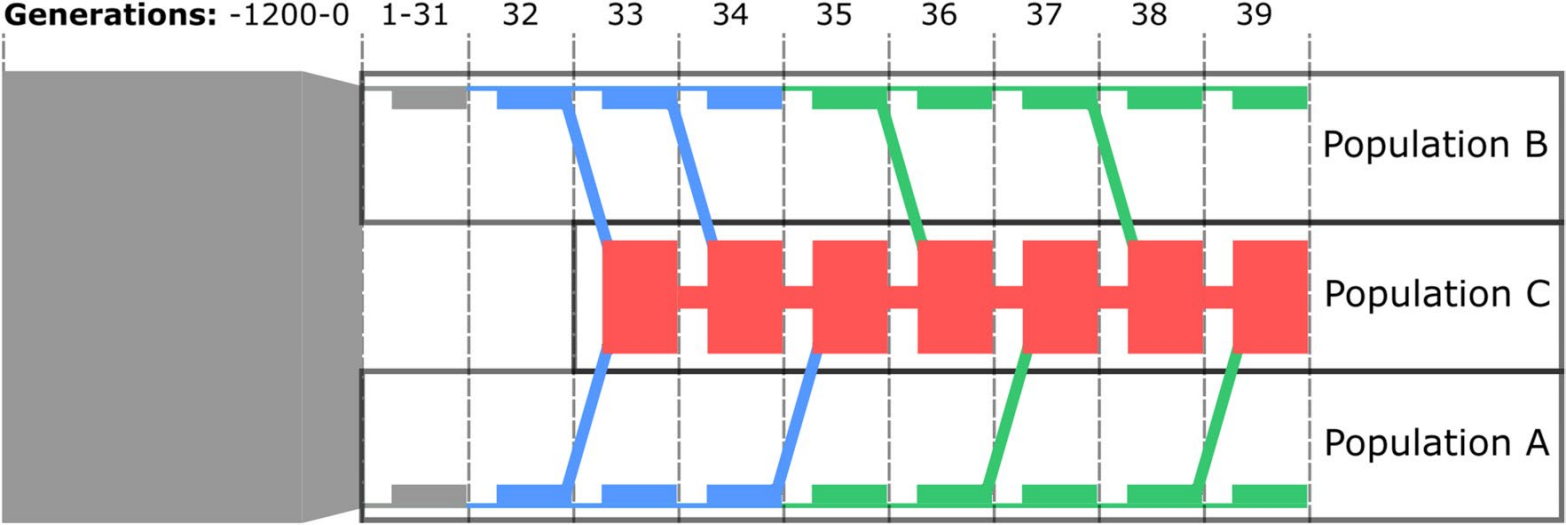
General population structures. Colors: Types of information made available for prediction. Grey: No information. Blue: Pedigree information. Red: Pedigree information and phenotypes. Green: Pedigree information and genomic information

A two-way-rotational crossbreeding system and genomic architecture were simulated with the QMSim software [16]. For all populations, generations did not overlap, the numbers of males and females were equal, sires and dams were chosen at random (no selection), mating was random and sampled without replacement, and the litter size was 6. We simulated 100 replicates. The population structures are shown in Fig. 1.

#### Historical population

The first generation in the historical population consisted of 3000 animals. The population size was constant for 1000 generations, and over the following 200 generations the population size decreased linearly to 2800 animals at the end.

#### Purebred populations

We created two purebred populations. Each purebred population was founded by 25 males and 25 females from the last generation in the historical population. The sampling of founders was random and independent for the two purebred populations. The purebred populations were kept separate for 39 generations. At each generation, 25 randomly selected sires were mated with 25 randomly selected dams; i.e., the effective population sizes were approximately 50 [17], and not all animals produced offspring. In the following, the two purebred populations are referred to as Population A and Population B.

#### Crossbred population

The first crossbred generation was founded by mating 75 males from Population A and 75 females from Population B. These animals were drawn from generation 32 of their respective population. The first generation in the crossbred population is referred to as generation 33. For generations 34 to 39, crossbred animals were created by mating 75 males from one of the purebred populations with 150 females from the crossbred population; i.e., for these generations, each purebred sire was mated with two crossbred dams. Sires were from Population A in odd-numbered generations and Population B in even-numbered generations. In the following, the crossbred population is referred to as Population C.

#### Genomic architecture

The genome consisted of five 100-cM chromosomes. Each chromosome contained 3500 markers and 350 quantitative trait loci (QTL). Marker positions, QTL positions, and allele frequencies were randomly and uniformly distributed. Marker and QTL genotypes were initialized in the first generation of the historical population.

On average, 12,104 of the 17,500 markers segregated with a minor allele frequency (MAF) higher than 0.01 in generation 32 of either purebred population. Meanwhile, 13,769 markers segregated with a MAF higher than 0.01 when marker genotypes were pooled across the purebred populations. Similarly, 1223 of the 1750 QTL segregated in generation 32 of either purebred population and 1394 QTL segregated when QTL genotypes were pooled across the purebred populations.

#### Genetic effects

We simulated both additive and dominant QTL effects. Additive and dominant QTL effects were identical across populations.

The additive genetic animal effects were solely based on additive QTL effects. The absolute additive QTL effects were drawn from a gamma-distribution with the standard parameters in QMSim [16]. Additive QTL effects were scaled by QMSim such that the additive genetic animal variance was 0.2 after the historical population [16].

The dominant QTL effects, were simulated as described by Wellmann and Bennewitz [18]:

1 $$\begin{aligned} \mathbf{d }^Q=\mathbf{h } \circ |{\varvec{\upbeta }}_1 -{\varvec{\upbeta }}_2| \text {,} \end{aligned}$$

where **d**$^Q$ is a vector of dominant QTL effects; $\mathbf{h }\sim {\mathcal {N}}(\frac{1}{2}\mathbf{1 },\frac{1}{10}\mathbf{I })$ is a vector of dominance degrees; $\circ$ is the Hadamard product; ${\varvec{\upbeta }}_1$ is a vector of additive QTL effects of the first QTL-allele; and ${\varvec{\upbeta }}_2$ is a vector of additive QTL effects of the second QTL-allele. Dominant genetic animal effects, **d**, were calculated as the sum of dominant QTL effects where the animal was heterozygous. Dominant genetic animal effects were scaled such that the dominant genetic animal variance was 0.1 in Population C. On average, 8% of the loci showed overdominance; 45% showed partial dominance that was greater than half the allele substitution effect; and 46% showed partial dominance that was less than half the allele substitution effect.

#### Phenotypes

We defined two phenotypes: a phenotype without dominance effects, $\mathbf{y }_{A}=\mathbf{a } + \mathbf{e }$, and a phenotype with dominance effects, $\mathbf{y }_{AD}=\mathbf{a } + \mathbf{d } + \mathbf{e }$, where **a** is a vector of additive genetic animal effects, **d** is a vector of dominant genetic animal effects, **e** is a vector of environmental effects, and $\mathbf{e } \sim {\mathcal {N}}(\mathbf{0 },0.8\mathbf{I })$. Note that $\mathbf{y }_{A}$ and $\mathbf{y }_{AD}$ have different narrow-sense heritabilities: $h^2_A=0.2$ and $h^2_{AD}=0.2/1.1$.

#### Information used for prediction

We used the information such as to represent a system where only crossbred animals were phenotyped and only the purebred animals were genotyped (Fig. 1). It is common practice to not genotype crossbred animals because it is more important to genotype selection candidates than phenotyped animals [19].

More specifically, pedigree information was kept only for animals born in generations 32 through 39. Animals born before generation 32 were regarded as unknown. Marker information was only available for purebred animals born in generations 35 through 39. Phenotypes were only available for crossbred animals born in generations 33 through 39.

### Prediction

#### General

We compared four methods for multibreed relationship matrices, i.e., the NRM [8]; García-Cortés and Toro (GT) [9]; Strandén and Mäntysaari (SM) [12]; and Legarra et al. (MF) [14]. All four methods can be extended to include genomic information using the single-step procedure [13, 20, 21]. For each method, we describe the theory, pedigree(s), incorporation of genomic information, the statistical model, and calculation of predicted breeding values. In Appendix 1, each method is showcased with a small example pedigree. We highly recommend readers who are unfamiliar with the methods to view Appendix 1 after reading their respective sections in Methods.

#### The NRM method

This method can be used for multibreed analyses in multiple ways. We use the NRM method such that we have one relationship matrix per breed. This allows us to partition the breeding values of crossbred animals into one term per purebred population. Furthermore, it allows the additive genetic variances to differ between purebred populations. In this study, the NRM method required two relationship matrices; one for terms contributed from Population A, and one for terms contributed from population B.

The recursive algorithm for each of the NRM matrices is:

2 $$\begin{aligned} a_{ij}= {\left\{ \begin{array}{ll} 1+\frac{1}{2}a_{sd}, &{} i=j\\ \frac{1}{2}(a_{is}+a_{id}), &{} \text {otherwise} , \end{array}\right. } \end{aligned}$$

where $i$ and $j$ denote animals, $a_{ij}$ is the pedigree-based covariance between the additive genetic effects of animals *i* and *j*, $s$ is the sire of $j$, and $d$ is the dam of $j$ [8].

The pedigrees for the two relationship matrices were different. The pedigree for Population A included animals from both Population A and Population C, and the pedigree for Population B included animals from both Population B and Population C. To create the pedigree for Population A, animals from Population B were removed from the pedigree and vice versa.

We used two genomic relationship matrices for the NRM method; $\mathbf{G }^{NRM}_A$ and $\mathbf{G }^{NRM}_B$. Preliminary genomic relationship matrices, $\mathbf{G }^{VanRaden}_A$ and $\mathbf{G }^{VanRaden}_B$, were calculated using VanRaden’s first method [22], genotypes from purebred animals in generations 35 to 39, and marker allele frequencies in the respective purebred base-populations. When calculating $\mathbf{G }^{VanRaden}_A$ and $\mathbf{G }^{VanRaden}_B$, a marker was included if its minor allele frequency was higher than 0.01 in its respective purebred base-population. The positive definiteness of genomic relationship matrices was ensured by using the weighted average between VanRaden’s first method and the sub-matrix of genotyped animals from its respective pedigree-based relationship matrix: $\mathbf{G }_X^{NRM}=0.05\{\mathbf{A }_X^{NRM}\}_{22}+0.95\mathbf{G }_X^{VanRaden}$, where $X \in \{A,B\}$ denotes the population and $\{\mathbf{A }_X^{NRM}\}_{22}$ is the sub-matrix of genotyped animals from the respective pedigree-based relationship matrix. The genomic relationship matrices, $\mathbf{G }_A^{NRM}$ and $\mathbf{G }_B^{NRM}$, were scaled and centered such that their average diagonal and off-diagonal elements were equal to those of the sub-matrices of genotyped animals from their respective pedigree-based relationship matrices. We calculated combined relationship matrices for genotyped and non-genotyped animals [20, 21] because some animals were not genotyped. The combined relationship matrices for genotyped and non-genotyped animals were $\mathbf{H }_A^{NRM}$ and $\mathbf{H }_B^{NRM}$ for animals with genetic contributions from Populations A and B, respectively.

The statistical model for the NRM method was:

3 $$\begin{aligned} \mathbf{y }=\mathbf{Xb} +\mathbf{Z }_A\mathbf{a }_A+\mathbf{Z }_B\mathbf{a }_B+\mathbf{e }, \end{aligned}$$

where **y** is a vector of phenotypes; **b** is a vector of parameters for the general mean, pedigree-derived breed proportion, and pedigree-derived heterosis; $\mathbf{a }_A$ is a vector of additive genetic effects from Population A; $\mathbf{a }_B$ is a vector of additive genetic effects from Population B; $\mathbf{e }$ is a vector of residuals; and $\mathbf{X }$, $\mathbf{Z }_A$, and $\mathbf{Z }_B$ are design matrices.

The three vectors with random effects (**a**$_A$, **a**$_B$, and $\mathbf{e }$) were assumed to be distributed as:

4 $$\begin{aligned} \begin{bmatrix} \mathbf{a }_A\\ \mathbf{a }_B\\ \mathbf{e } \end{bmatrix} \sim {\mathcal {N}} \begin{pmatrix} \begin{bmatrix} \mathbf{0 }\\ \mathbf{0 }\\ \mathbf{0 } \end{bmatrix} , \begin{bmatrix} \mathbf{A }^{NRM}_{A}\sigma ^2_{A_A} &{} &{} &{} \\ \mathbf{0 } &{} \mathbf{A }^{NRM}_{B}\sigma ^2_{A_B} &{} &{} \\ \mathbf{0 } &{} \mathbf{0 } &{} \mathbf{I }\sigma _e^2 \end{bmatrix} \end{pmatrix}, \end{aligned}$$

where $\mathbf{A }^{NRM}_{A}$ is a relationship matrix for additive genetic effects from Population A; $\mathbf{A }^{NRM}_{B}$ is a relationship matrix for additive genetic effects from Population B; $\sigma ^2_{A_{A}}$ is the additive genetic variance in Population A; $\sigma ^2_{A_{B}}$ is the additive genetic variance in Population B; **0**s are vectors or matrices of zeros; **I** is an identity matrix; and $\sigma ^2_e$ is the residual variance. For prediction with genomic information, $\mathbf{A }^{NRM}_{A}$ was replaced with $\mathbf{H }^{NRM}_{A}$ and $\mathbf{A }^{NRM}_{B}$ was replaced with $\mathbf{H }^{NRM}_{B}$.

The vector of predicted breeding values for the NRM method was:

5 $$\begin{aligned} \mathbf{ebv} ^{NRM}= \begin{bmatrix} \{\hat{\mathbf{a }}_A\}_P \\ \mathbf{0 } \\ \{\hat{\mathbf{a }}_A\}_C \end{bmatrix} + \begin{bmatrix} \mathbf{0 } \\ \{\hat{\mathbf{a }}_B\}_P \\ \{\hat{\mathbf{a }}_B\}_C \end{bmatrix} , \end{aligned}$$

where $\hat{\mathbf{a }}_A$ and $\hat{\mathbf{a }}_B$ are the vectors of predicted additive genetic effects in the statistical model for the NRM method (Eq. 4); subscript *P* denotes that the sub-vector only contains predicted effects from purebred animals; subscript *C* denotes that the sub-vector only contains predicted effects from crossbred animals; and **0**s are vectors of zeros.

#### The GT method

This method partitions the additive genetic relationship into several partial relationship matrices [9]: one for each breed (partial relationship matrices for breed-specific terms; $\mathbf{A }^{GT}_{A}$ and $\mathbf{A }^{GT}_{B}$ in our study), and one for each pair of breeds (partial relationship matrices for segregation terms; $\mathbf{A }^{GT}_{AB}$ in our study). The partial relationship matrix for segregation terms captures the increase in additive genetic variance in crossbred animals [4, 9].

The recursive algorithm for calculating $\mathbf{A }^{GT}_{A}$ is [9]:

6 $$\begin{aligned} a_{ij}= {\left\{ \begin{array}{ll} f^A_i+\frac{1}{2}a_{sd}, \quad &{} i=j\\ \frac{1}{2}(a_{is}+a_{id}), \quad &{} \text {otherwise} , \end{array}\right. } \end{aligned}$$

where $i$, $j$, $s$, and $d$ are as described for the algorithm for the NRM method (Eq. 2); $a_{ij}$ is the pedigree-based covariance between the breed-specific partial additive genetic effects of animals *i* and *j*; and $f^A_i$ is the proportion of genetic material from Population A in animal $i$. The sub-matrix of $\mathbf{A }^{GT}_{A}$ for purebred animals is identical to its analogous sub-matrix of $\mathbf{A }^{NRM}_{A}$.

The recursive algorithm for $\mathbf{A }^{GT}_{AB}$ is:

7 $$\begin{aligned} a_{ij}= {\left\{ \begin{array}{ll} 2\left( f^{A}_sf^{B}_s+f^{A}_df^{B}_d\right) +\frac{1}{2}a_{sd}, \quad &{} i=j\\ \frac{1}{2}(a_{is}+a_{id}), \quad &{} \text {otherwise} , \end{array}\right. } \end{aligned}$$

where $i$, $j$, $s$, and $d$ are as for the algorithm for the NRM method (Eq. 2); $a_{ij}$ is the pedigree-based covariance between the additive genetic segregation effects of animals *i* and *j*; $f^A_s$ and $f^B_s$ are the proportions of genetic material from Population A and Population B, respectively, in the sire of animal $j$; and $f^A_d$ and $f^B_d$ are the proportions of genetic material from Population A and Population B, respectively, in the dam of animal $j$. Both diagonal and off-diagonal elements in $\mathbf{A }^{GT}_{AB}$ can only be non-zero for descendants of crossbred animals.

The pedigree for the GT method included all the animals, purebred and crossbred, in generations 32 through 39.

We used two genomic relationship matrices for the GT method; $\mathbf{G }^{GT}_A$ and $\mathbf{G }^{GT}_B$. Generally, the single-step procedure for the GT method requires that marker alleles are phased and traced such that their breed of origin can be determined [13]; however, tracing the breed of origin of alleles was not required in this study because we only used genotypes from purebred animals. Therefore, $\mathbf{G }^{GT}_A$ and $\mathbf{G }^{GT}_B$ were the same as the genomic relationship matrices for the NRM method; i.e., $\mathbf{G }^{GT}_A=\mathbf{G }^{NRM}_A$ and $\mathbf{G }^{GT}_B=\mathbf{G }^{NRM}_B$. The single-step procedure [20, 21] was used for the partial relationship matrices for breed-specific terms. The combined partial relationship matrices for breed-specific terms for genotyped and non-genotyped animals were $\mathbf{H }_A^{GT}$ and $\mathbf{H }_B^{GT}$ for animals with genetic contributions from Populations A and B, respectively. The partial relationship matrix for the segregation term did not include genomic information.

The statistical model for the GT method was:

8 $$\begin{aligned} \mathbf{y }=\mathbf{Xb} +\mathbf{Z }_A\mathbf{a }_A+\mathbf{Z }_B\mathbf{a }_B+\mathbf{Z }_{AB}\mathbf{a }_{AB}+\mathbf{e }, \end{aligned}$$

where **y**, **b**, and **X** are as described for the statistical model for the NRM method (Eq. 4); $\mathbf{a }_A$ is a vector of breed-specific partial additive genetic effects from Population A; $\mathbf{a }_B$ is a vector of breed-specific partial additive genetic effects from Population B; $\mathbf{a }_{AB}$ is a vector of additive genetic segregation effects between Populations A and B; $\mathbf{e }$ is a vector of residuals; and $\mathbf{Z }_A$, $\mathbf{Z }_B$, and $\mathbf{Z }_{AB}$ are design matrices.

The four vectors of random effects (**a**$_A$, **a**$_B$, **a**$_{AB}$ and $\mathbf{e }$) were assumed to be distributed as:

9 $$\begin{aligned} \begin{bmatrix} \mathbf{a }_A\\ \mathbf{a }_B\\ \mathbf{a }_{AB}\\ \mathbf{e } \end{bmatrix} \sim {\mathcal {N}} \begin{pmatrix} \begin{bmatrix} \mathbf{0 }\\ \mathbf{0 }\\ \mathbf{0 }\\ \mathbf{0 } \end{bmatrix} ,\begin{bmatrix} \mathbf{A }^{GT}_A\sigma ^2_{A_A} &{} &{} &{} \\ \mathbf{0 } &{} \mathbf{A }^{GT}_B\sigma ^2_{A_B} &{} &{} \\ \mathbf{0 } &{} \mathbf{0 } &{} \mathbf{A }^{GT}_{AB}\sigma ^2_{A_{AB}} &{} \\ \mathbf{0 } &{} \mathbf{0 } &{} \mathbf{0 } &{} \mathbf{I }\sigma_e ^2 \end{bmatrix} \end{pmatrix}, \end{aligned}$$

where $\mathbf{A }^{GT}_{A}$ is a partial relationship matrix for the breed-specific term from Population A; $\mathbf{A }^{GT}_{B}$ is a partial relationship matrix for the breed-specific term from Population B; $\mathbf{A }^{GT}_{AB}$ is a partial relationship matrix for the segregation term between Populations A and B; $\sigma ^2_{A_{A}}$ is the additive genetic variance in Population A; $\sigma ^2_{A_{B}}$ is the additive genetic variance in Population B; $\sigma ^2_{A_{AB}}$ is the segregation variance between Populations A and B; **0**s are vectors or matrices of zeros; **I** is an identity matrix; and $\sigma ^2_e$ is the residual variance. For prediction with genomic information, $\mathbf{A }^{GT}_{A}$ was replaced with $\mathbf{H }^{GT}_{A}$ and $\mathbf{A }^{GT}_{B}$ was replaced with $\mathbf{H }^{GT}_{B}$.

The vector of predicted breeding values for the GT method was:

10 $$\begin{aligned} \mathbf{ebv} ^{GT}= \begin{bmatrix} \{\hat{\mathbf{a }}_A\}_P \\ \mathbf{0 } \\ \{\hat{\mathbf{a }}_A\}_{C:F1} \\ \{\hat{\mathbf{a }}_A\}_{C:R} \end{bmatrix} + \begin{bmatrix} \mathbf{0 } \\ \{\hat{\mathbf{a }}_B\}_P \\ \{\hat{\mathbf{a }}_B\}_{C:F1} \\ \{\hat{\mathbf{a }}_B\}_{C:R} \end{bmatrix} + \begin{bmatrix} \mathbf{0 } \\ \mathbf{0 } \\ \mathbf{0 } \\ \hat{\mathbf{a }}_{AB} \end{bmatrix} , \end{aligned}$$

where $\hat{\mathbf{a }}_A$, $\hat{\mathbf{a }}_B$, $\hat{\mathbf{a }}_{AB}$ are the vectors of predicted partial additive genetic effects in the statistical model for the GT method (Eq. 8); subscript *P* denotes that the sub-vector only contains the predicted effects from purebred animals; subscript *C:F1* denotes that the sub-vector only contains the predicted effects from F1 crossbred animals; subscript *C:R* denotes that the sub-vector only contains the predicted effects from rotationally crossbred animals; and **0**s are vectors of zeros.

#### The SM method

This method is an approximation of the GT method and it partitions the additive genetic variance in the same way.

The relationship matrices for the SM method are calculated as:

11 $$\begin{aligned} \mathbf{A }^{GT}_{A}\approx & {} \mathbf{A }^{SM}_A=\mathbf{F }_A\mathbf{A }^{NRM}_A\mathbf{F }_A, \end{aligned}$$

12 $$\begin{aligned} \mathbf{A }^{GT}_{B}\approx & {} \mathbf{A }^{SM}_B=\mathbf{F }_B\mathbf{A }^{NRM}_B\mathbf{F }_B, \end{aligned}$$

13 $$\begin{aligned} \mathbf{A }^{GT}_{AB}\approx & {} \mathbf{A }^{SM}_{AB}=\mathbf{F }_{AB}\mathbf{A }^{NRM}_{AB}\mathbf{F }_{AB}, \end{aligned}$$

where $\mathbf{F }_A$ and $\mathbf{F }_B$ are diagonal matrices with square roots of breed proportions for populations A and B, respectively; $\mathbf{A }^{NRM}_{AB}$ is a NRM-based relationship matrix representing at least all the descendants of crossbred animals; and $\mathbf{F }_{AB}$ is a diagonal matrix with square roots of the “$2\left( \mathbf{f }^{A}_s\mathbf{f }^{B}_s + \mathbf{f }^{A}_d\mathbf{f }^{B}_d\right)$” term from Eq. 7. As for the GT method, the sub-matrices of $\mathbf{A }^{SM}_{A}$ and $\mathbf{A }^{SM}_{B}$ for purebred animals are identical to submatrices from $\mathbf{A }^{NRM}_{A}$ and $\mathbf{A }^{NRM}_{B}$ for purebreds animals, respectively.

The SM method is equivalent to random-regressions of additive genetic effects on $\mathbf{F }_A,$ $\mathbf{F }_B$, and $\mathbf{F }_{AB}$, respectively [12], because $\mathbf{F }_A\mathbf{a }_A \sim {\mathcal {N}}\left( \mathbf{0 }, \mathbf{F }_A\mathbf{A }^{NRM}_{A}\mathbf{F }_A^T\right) , \mathbf{F }_B\mathbf{a }_B \sim {\mathcal {N}}\left( \mathbf{0 }, \mathbf{F }_B\mathbf{A }^{NRM}_{B}\mathbf{F }_B^T\right) ,$ and $\mathbf{F }_{AB}\mathbf{a }_{AB} \sim {\mathcal {N}}\left( \mathbf{0 }, \mathbf{F }_{AB}\mathbf{A }^{NRM}_{AB}\mathbf{F }_{AB}^T\right)$. In this study, we apply the SM method through random-regression.

Three pedigrees were constructed for the SM method: one for each purebred population, which are identical to those for the NRM method, and the third is for the partial relationship matrix for the segregation term between Populations A and B. The partial relationship matrix for the segregation term between Populations A and B was calculated with a pedigree from which all purebred and F1 animals had been removed.

We did not use the same pedigree for segregation effects as described by the SM method [12]. They used the full pedigree to construct an additive genetic relationship matrix on which they applied random regression. However, using the full pedigree may promote discreprancies between the GT and SM methods. According to the GT method, segregation effects are independent among all offspring from F1 animals and their magnitude only depend on the breed proportions of parental animals. For the SM method, a deep pedigree for segregation effects would increase the likelihood of both non-zero inbreeding coefficients in offspring from F1 animals and covariance between offspring from F1 animals. Therefore, the compliance between the GT and SM methods should be greater if purebred and F1 animals are removed from the pedigree for segregation effects, as done in this study.

The genomic relationship matrices for this method were the same as for both the NRM and GT methods. As for the GT method, we calculated combined relationship matrices for genotyped and non-genotyped animals for the breed-specific terms but not for the segregation term.

The statistical model for the SM method was:

14 $$\begin{aligned} \mathbf{y }=\mathbf{Xb} +\mathbf{Z }_A\mathbf{F }_A\mathbf{a }_A+\mathbf{Z }_B\mathbf{F }_B\mathbf{a }_B+\mathbf{Z }_{AB}\mathbf{F }_{AB}\mathbf{a }_{AB}+\mathbf{e }, \end{aligned}$$

where $\mathbf{F }_{A}$, $\mathbf{F }_{B}$, and, $\mathbf{F }_{AB}$ are as defined for the calculation of partial relationship matrices with the SM method (Eqs. 11, 12, 13); and the remaining components are the same as in the statistical model for the GT method (Eq. 8). Note that the additive genetic vectors now consist of regression coefficients.

The four vectors of random effects (**a**$_A$, **a**$_B$, **a**$_{AB}$ and $\mathbf{e }$) were assumed to be distributed as:

15 $$\begin{aligned} \begin{bmatrix} \mathbf{a }_A\\ \mathbf{a }_B\\ \mathbf{a }_{AB}\\ \mathbf{e } \end{bmatrix} \sim {\mathcal {N}} \begin{pmatrix} \begin{bmatrix} \mathbf{0 }\\ \mathbf{0 }\\ \mathbf{0 }\\ \mathbf{0 } \end{bmatrix} ,\begin{bmatrix} \mathbf{A }^{NRM}_A\sigma ^2_{A_A} &{} &{} &{} \\ \mathbf{0 } &{} \mathbf{A }^{NRM}_B\sigma ^2_{A_B} &{} &{} \\ \mathbf{0 } &{} \mathbf{0 } &{} \mathbf{A }^{NRM}_{AB}\sigma ^2_{A_{AB}} &{} \\ \mathbf{0 } &{} \mathbf{0 } &{} \mathbf{0 } &{} \mathbf{I }\sigma _e^2 \end{bmatrix} \end{pmatrix}, \end{aligned}$$

where $\sigma ^2_{A_{A}}$, $\sigma ^2_{A_{B}}$, $\sigma ^2_{A_{AB}}$, $\sigma ^2_{e}$, **0**, and **I** are as in the statistical model for the GT method (Eq. 8); $\mathbf{A }^{NRM}_{A}$ and $\mathbf{A }^{NRM}_{B}$ are as in the statistical model for the NRM method (Eq. 4); and $\mathbf{A }^{NRM}_{AB}$ is the usual numerator relationship matrix based on the pedigree without purebred and F1 animals. For prediction with genomic information, $\mathbf{A }^{NRM}_{A}$ was replaced with $\mathbf{H }^{NRM}_{A}$ and $\mathbf{A }^{NRM}_{B}$ was replaced with $\mathbf{H }^{NRM}_{B}$.

The vector of predicted breeding values for the SM method was:

16 $$\begin{aligned} \mathbf{ebv} ^{SM}= \begin{bmatrix} \{\hat{\mathbf{a }}_A\}_P \\ \mathbf{0 } \\ \{\hat{\mathbf{a }}_A\}_{C:F1} \\ \{\hat{\mathbf{a }}_A\}_{C:R} \end{bmatrix} + \begin{bmatrix} \mathbf{0 } \\ \{\hat{\mathbf{a }}_B\}_P \\ \{\hat{\mathbf{a }}_B\}_{C:F1} \\ \{\hat{\mathbf{a }}_B\}_{C:R} \end{bmatrix} + \begin{bmatrix} \mathbf{0 } \\ \mathbf{0 } \\ \mathbf{0 } \\ \hat{\mathbf{a }}_{AB} \end{bmatrix} , \end{aligned}$$

where $\hat{\mathbf{a }}_A$, $\hat{\mathbf{a }}_B$, $\hat{\mathbf{a }}_{AB}$ are the vectors of predicted partial additive genetic effects in the statistical model for the SM method (Eq. 14); and **0**, subscript *P*, subscript *C:F1*, and subscript *C:R* are as defined for the GT method (Eq. 10).

### The MF method

This method is conceptually different from the other methods. The other methods model populations as separate entities while the MF method models populations as sub-populations derived from a common ancestral population. In practice, this is done by identifying each sub-population through a metafounder, calculating an additive genetic relationship matrix, $\varvec{\Gamma }$, between metafounders, and then incorporating this information into one shared additive genetic relationship matrix for all populations, $\mathbf{A }\left( \varvec{\Gamma }\right)$. In theory, this method should simultaneously account for both the breed-specific terms and the segregation term [14].

The metafounder relationships can be calculated in several ways [14, 23]. We used the method proposed by Garcia-Baccino et al. [23]:

17 $$\begin{aligned} \varvec{\Gamma } = \begin{bmatrix} \gamma _{A} &{} \\ \gamma _{AB} &{} \gamma _{B} \\ \end{bmatrix} = 8 \begin{bmatrix} \sigma ^2_{p^*_A} &{} \\ \sigma _{p^*_{A}p^*_{B}} &{} \sigma ^2_{p^*_B} \\ \end{bmatrix}, \end{aligned}$$

where $\gamma _{A}$ is the metafounder relationship for Population A; $\gamma _{B}$ is the metafounder relationship for Population B; $\gamma _{AB}$ is the metafounder relationship between Populations A and B; $\sigma ^2_{p^*_A}$ is the variance of marker allele frequencies in Population A; $\sigma ^2_{p^*_B}$ is the variance of marker allele frequencies in Population B; $\sigma _{p^*_{A}p^*_{B}}$ is a covariance between marker allele frequencies in Populations A and B; $p^*_A$ and $p^*_B$ are marker-allele frequencies in the base populations of Populations A and B, respectively; and the asterisk superscripts in $p^*_A$ and $p^*_B$ denote that allele annotations were randomized such that ${\mathbb {E}}(p^*_A)={\mathbb {E}}(p^*_B)=\frac{1}{2}$.

In this study, metafounder relationships were calculated with estimated marker allele frequencies in generation 32. We estimated marker allele frequencies as proposed by Gengler et al. [24] and genotypes from purebred animals in generations 35 to 39. Marker allele frequencies were estimated independently for each purebred population. Finally, metafounder relationships were calculated from markers that have a minor allele frequency higher than 0.01 when averaged across the purebred base-populations. The average metafounder relationship matrix across replicates, $\bar{\varvec{\Gamma }}$, was: $$\bar{\varvec{\Gamma }} = \begin{bmatrix} \bar{\gamma _{A}} &{} \\ \bar{\gamma _{AB}} &{} \bar{\gamma _{B}} \\ \end{bmatrix} = \begin{bmatrix} 0.80 &{} \\ 0.38 &{} 0.80 \\ \end{bmatrix}$$.

The recursive algorithm for the MF method is:

18 $$\begin{aligned} a_{ij}= {\left\{ \begin{array}{ll} 1+\frac{1}{2}\gamma _{A}, &{} i=j \wedge i \in \mathbf{m }_A\\ 1+\frac{1}{2}\gamma _{B}, &{} i=j \wedge i \in \mathbf{m }_B\\ \gamma _{A}, &{} i \ne j \wedge \{i,j\} \subset \mathbf{m }_A\\ \gamma _{B}, &{} i \ne j \wedge \{i,j\} \subset \mathbf{m }_B\\ \gamma _{AB}, &{} i \ne j \wedge \left[ (i \in \mathbf{m }_A \wedge j \in \mathbf{m }_B) \vee (i \in \mathbf{m }_B \wedge j \in \mathbf{m }_A) \right] \\ 1+\frac{1}{2}a_{sd}, &{} i=j \wedge i \not \in \{\mathbf{m }_A,\mathbf{m }_B\}\\ \frac{1}{2}(a_{is}+a_{id}), &{} \text {otherwise}, \end{array}\right. } \end{aligned}$$

where $i$, $j$, $s$, and $d$ are as in the recursive algorithms for the NRM and GT methods (Eqs. 4 and 8); $a_{ij}$ is as in the recursive algorithm for the NRM method (Eq. 4); $\gamma _{A}$, $\gamma _{B}$, and $\gamma _{AB}$ are the metafounder relationships (Eq. 17); $\mathbf{m }_A$ is a vector of base animals from Population A; $\mathbf{m }_B$ is a vector of base animals from Population B; $\wedge$ is the logical “*and*”; and $\vee$ is the logical “*or*”. Please note that the last two elements of the recursive algorithm for the MF method are the same as in the algorithm for the NRM method. In other words, the only differences between the NRM method and the MF method are that base animals are related and their inbreeding coefficient can be greater than zero. These differences then carry over into the additive genetic relationships for animals which are not in the base population.

The pedigree for the MF method included all the animals, purebred and crossbred, in generations 32 to 39.

The MF method uses one genomic relationship matrix across all populations; $\mathbf{G }^{MF}$. A preliminary genomic relationship matrix, $\mathbf{G }^{VanRaden}$, was calculated using VanRaden’s first method [22], and genotypes from purebred animals in generations 35 to 39; however, we scaled and centered the genomic relationship matrix with allele frequencies of 0.5. Markers were included in the genomic relationship matrix if their minor allele frequency was higher than 0.01 when pooling genotypes from the purebred base-populations. The positive definiteness of the genomic relationship matrix was ensured by using the weighted average of $\mathbf{G }^{VanRaden}$ and the sub-matrix of genotyped animals from the pedigree-based relationship matrix: $\mathbf{G }^{MF}=0.05\{\mathbf{A }(\varvec{\Gamma })\}_{22}+0.95\mathbf{G }^{VanRaden}$, where $\{\mathbf{A }(\varvec{\Gamma })\}_{22}$ is the sub-matrix of genotyped animals from the pedigree-based relationship matrix. The genomic relationship matrix, $\mathbf{G }^{MF}$, was not scaled and centered such that its average diagonal and off-diagonal elements were equal to those of $\{\mathbf{A }(\varvec{\Gamma })\}_{22}$ because $\mathbf{G }^{MF}$ and $\{\mathbf{A }(\varvec{\Gamma })\}_{22}$ are comparable when $\mathbf{G }^{MF}$ and $\varvec{\Gamma }$ are calculated with the same set of markers. We calculated a combined relationship matrix for genotyped and non-genotyped animals [14, 20, 21], $\mathbf{H }(\varvec{\Gamma })$, because some animals were not genotyped.

The statistical model for the MF method was:

19 $$\begin{aligned} \mathbf{y }=\mathbf{Xb} +\mathbf{Za} +\mathbf{e }, \end{aligned}$$

where **y**, **b**, and **X** are as described for the NRM method (Eq. 4); $\mathbf{a }$ is a vector of additive genetic effects; $\mathbf{e }$ is a vector of residuals; and **Z** is a design matrix.

The two vectors of random effects (**a** and $\mathbf{e }$) were distributed as:

20 $$\begin{aligned} \begin{bmatrix} \mathbf{a }\\ \mathbf{e } \end{bmatrix} \sim {\mathcal {N}} \begin{pmatrix} \begin{bmatrix} \mathbf{0 }\\ \mathbf{0 } \end{bmatrix} , \begin{bmatrix} \mathbf{A }(\varvec{\Gamma })\sigma ^2_{A_{MF}} &{} \\ \mathbf{0 } &{} \mathbf{I }\sigma _e^2 \end{bmatrix} \end{pmatrix}, \end{aligned}$$

where **A**($\varvec{\Gamma }$) is the additive relationship matrix with metafounders [14]; $\varvec{\Gamma }$ is the additive relationship matrix between metafounders; $\sigma ^2_{A_{MF}}$ is the additive genetic variance in the ancestral population; **0**s are vectors or matrices of zeros; **I** is an identity matrix; and $\sigma ^2_e$ is the residual variance. The additive genetic relationship matrix, $\mathbf{A }(\varvec{\Gamma })$, was replaced with $\mathbf{H }(\varvec{\Gamma })$ when breeding values were predicted with genomic prediction.

The vector of predicted breeding values for the MF method was:

21 $$\begin{aligned} \mathbf{ebv} ^{MF}=\hat{\mathbf{a }}, \end{aligned}$$

where $\hat{\mathbf{a }}$ is the vector of predicted additive genetic effects in the statistical model for the MF method (Eq. 19).

#### Variance components

We estimated variance components for each method and its respective statistical model (Eqs. 4, 8, 14, and 19). Variance components were only estimated with pedigree information. Breeding values were predicted with these estimated variance components regardless of whether breeding values were predicted with or without genomic information.

The estimated variance components for the phenotype without dominance effects are in Table 1. For presentation only, the estimated additive genetic variance from the MF method was transformed using the estimated metafounder relationships, $\varvec{\Gamma }$, such that the parametrization was the same as for the GT method [14]:

22 $$\begin{aligned} \begin{aligned} \sigma ^2_{A_A}&=\sigma ^2_{A_{MF}}\left( 1-\frac{1}{2}\gamma _{A}\right) \\ \sigma ^2_{A_B}&=\sigma ^2_{A_{MF}}\left( 1-\frac{1}{2}\gamma _{B}\right) \\ \sigma ^2_{A_{AB}}&=\frac{1}{8}\sigma ^2_{A_{MF}}\left( \gamma _{A} + \gamma _{B} - 2\gamma _{AB}\right) \\ \end{aligned} {,} \end{aligned}$$

where $\sigma ^2_{A_{A}}$, $\sigma ^2_{A_{B}}$, and $\sigma ^2_{A_{AB}}$ are the partial additive genetic variance components; $\sigma ^2_{A_{MF}}$ is the estimated additive genetic variance in the ancestral population (Eq. 20); and $\gamma _{A}$, $\gamma _{B}$, and $\gamma _{AB}$ are metafounder relationships (Eq. 8).

**Table 1:** Means and standard deviations of variance components across replicates

Method	$\sigma^{2}_{A_A}$	$\sigma^{2}_{A_B}$	$\sigma^{2}_{A_{AB}}$	$\sigma^2_{e}$
True	0.15 ± 0.01	0.15 ± 0.01	0.023 ± 0.003	0.80^a^
GT	0.15 ± 0.04	0.14 ± 0.04	0.035 ± 0.039	0.80 ± 0.02
MF	0.15 ± 0.02	0.15 ± 0.02	0.026 ± 0.005	0.80 ± 0.02
SM	0.25 ± 0.07	0.19 ± 0.06	0.297 ± 0.052	0.61 ± 0.04
NRM	0.23 ± 0.05	0.22 ± 0.05		0.51 ± 0.07

$\sigma ^2_{A_A}$: Additive genetic variance for Population A

$\sigma ^2_{A_B}$: Additive genetic variance for Population B

$\sigma ^2_{A_{AB}}$: Additive genetic segregation variance between populations A and B

$$\sigma ^2_{e}$$: Residual variance

^a^The true residual variance was constant across replicates

We calculated true partial additive genetic variance components and used them as reference for the magnitude of the estimated variance components in Table 1. The true partial additive genetic variance components were calculated with the parametrization of the GT method and the phenotype without dominance effects.

The true partial additive genetic variance for breed-specific effects from Population A was calculated as:

23 $$\begin{aligned} \sigma ^2_{A_A}= 2\sum _{i=1}^{n_{qtl}} p_{i,A}\left( 1-p_{i,A}\right) \left( \upbeta _{i,1}-\upbeta _{i,2}\right) ^2, \end{aligned}$$

where $\sigma ^2_{A_A}$ is the true partial additive genetic variance for breed-specific effects from Population A; $n_{qtl}$ is the number of QTL; $p_{i,A}$ is the allele frequency at QTL *i* in base animals from Population A; $\upbeta _{i,1}$ is the additive genetic effect of the first QTL allele at QTL *i*; and $\upbeta _{i,2}$ is the additive genetic effect of the second QTL allele at QTL *i*. The partial additive genetic variance for breed-specific effects from Population B was calculated in the same way.

The true partial additive genetic variance for segregation effects between Populations A and B was calculated as [4]:

24 $$\begin{aligned} \sigma ^2_{A_{AB}}= 2\sum _{i=1}^{n_{qtl}}\left[ p_{i,F1}\left( 1-p_{i,F1}\right) \left( \upbeta _{i,1} - \upbeta _{i,2} \right) ^2\right] - \frac{1}{2}\left[ \sigma ^2_{A_A} + \sigma ^2_{A_B}\right] \text {,} \end{aligned}$$

where $\upbeta _{i,1}$, $\upbeta _{i,2}$, and $n_{qtl}$ are as in Eq. 23; $\sigma ^2_{AB}$ is the true partial additive genetic variance for segregation effects between Populations A and B; $p_{i,F1}$ is a vector of QTL allele frequencies in generation 33 of Population C; $\sigma ^2_A$ is the true partial additive genetic variance for breed-specific effects from Population A; and $\sigma ^2_B$ is the true partial additive genetic variance for breed-specific effects from Population B.

#### Software for analysis and prediction

Most data-handling was carried out in the R-software [25]. The relationship matrices for the GT and MF methods were calculated using the RcppArmadillo R-package [26]. The relationship matrices for the NRM and SM methods were calculated using the DMU software [27]. Variance components were estimated using the AI-ReML algorithm in the DMU software package [27]. Additive genetic effects were predicted using the best linear unbiased prediction (BLUP) method and the Preconditioned Conjugate Gradient algorithm implemented in DMU software [27].

### Comparison of the methods

#### General

We compared how well the methods predicted accurate and unbiased breeding values in animals from generation 39. In the following, we describe how we calculated true breeding values, accuracies, and biases, and the statistical methods used to compare the methods. We stratified the comparison according to population.

#### True breeding values

The true breeding value depends on whether the phenotype includes only an additive genetic term, or both additive genetic and dominant genetic terms.

The true breeding values with only an additive genetic term were calculated as:

25 $$\begin{aligned} \mathbf{tbv} _{A} = \mathbf{Q }({\varvec{\upbeta }}_{1} - {\varvec{\upbeta }}_{2})+2\mathbf{J }{\varvec{\upbeta }}_{2}, \end{aligned}$$

where **Q** is a QTL genotype matrix with allelic loads of the first allele; ${\varvec{\upbeta }}_{1}$ is a vector of additive genetic effects of the first QTL allele; ${\varvec{\upbeta }}_{2}$ is a vector of additive genetic effects of the second QTL allele; and **J** is a matrix of 1s with dimensions equal those of **Q**.

In the presence of dominance, the true breeding value of an animal depends on its ability to promote both additive and dominance genetic effects in its offspring [28, 29]. Therefore, the true breeding value now depends on the genotypes of the mate. True breeding values with a dominance term can be calculated with allele frequencies from the population of mating candidates [28, 29]. The true breeding values with both an additive term and a dominance term were calculated as:

26 $$\begin{aligned} \mathbf{tbv} ^X_{AD} = \mathbf{tbv} _{A} + (\mathbf{Q }-\mathbf{J}) \left[ (\mathbf{1 }-2\mathbf{p }_{X}) \circ \mathbf{d }^Q\right] , \end{aligned}$$

where $X \in \{A,B,C\}$ denotes the population to which the possible mating candidates belong; **p**$_X$ is a vector of QTL allele frequencies in population *X*; **d**^*Q*^ is a vector of dominant QTL effects; $\circ$ is the Hadamard product; **1** is a vector of ones; and $\mathbf{tbv} _{A}$, **Q**, ${\varvec{\upbeta }}_{1}$, ${\varvec{\upbeta }}_{2}$, and **J** are as for true breeding values with only an additive genetic term (Eq. 25).

#### Accuracy and bias

We evaluated the methods according to their prediction accuracy and prediction bias. We used two measures for the prediction bias [30, 31]: level bias and dispersion bias.

The prediction accuracy was defined as Pearson’s correlation between true breeding values and predicted breeding values:

27 $$\begin{aligned} Accuracy=\rho (\mathbf{tbv} ,\mathbf{ebv} ), \end{aligned}$$

where $\rho (.)$ is the Pearson correlation function; **tbv** is a vector of true breeding values; and **ebv** is a vector of predicted breeding values.

The level bias was calculated as:

28 $$\begin{aligned} \mu _{bias} = \overline{\mathbf{ebv }} - \overline{\mathbf{tbv }} + \overline{\mathbf{tbv }}_{base}, \end{aligned}$$

where $\mu _{bias}$ is the level bias; $\overline{\mathbf{ebv }}$ is the mean predicted breeding value in validation animals; $\overline{\mathbf{tbv }}$ is the mean true breeding values in validation animals; and $\overline{\mathbf{tbv }}_{base}$ is the mean true breeding value in base animals. The correction for $\overline{\mathbf{tbv }}_{base}$ was required because the true breeding values, in contrast to predicted breeding values, differed from zero in the base populations. There is no level bias when $\mu _{bias}$ is equal to 0.

The dispersion bias was calculated as:

29 $$\begin{aligned} b_{bias}= \frac{cov(\mathbf{tbv} ,\mathbf{ebv} )}{var(\mathbf{ebv} )}, \end{aligned}$$

where $b_{bias}$ is the dispersion bias; $cov()$ is the empirical covariance; $\mathbf{ebv}$ is a vector of predicted breeding values; $\mathbf{tbv}$ is a vector of true breeding values; and $var()$ is the empirical variance. There is no dispersion bias when $b_{bias}$ is equal to 1.

#### Statistical analysis of accuracy, level bias, and dispersion bias

The accuracies and biases were compared across methods, use of genomic information, and replicates; but not across populations and definition of true breeding values.

We used non-parametric tests because accuracies and biases were heteroscedastic across methods and not normally distributed.

We investigated whether a method was more accurate or biased than others using paired Wilcoxon signed rank tests. We used paired tests to compare the methods to remove the variation caused by the stochastic simulation; i.e., the methods were paired within replicates. Furthermore, we investigated whether the methods were biased using the one-sample Wilcoxon signed rank tests. The null hypotheses for these tests were that the level biases were equal to 0 and that the dispersion biases were equal to 1.

We used the Bonferroni-correction of p-values to control for multiple testing: $\alpha _{bon}=\alpha / n_{tests} = 0.05/\text {1000}$, where $\alpha$ is the significance level and $n_{tests}$ is the number of statistical tests. Among the 1000 tests, $n_p \times n_g \times n_m \times \left( n_m - 1 \right) /2=\text {840}$ were comparisons between validation parameters and $(n_{p}-1) \times n_g \times n_m =\text {160}$ were tests for whether validation parameters differed from expected values, where $n_{p}=3$ is the number of validation parameters (accuracy, level bias, and dispersion bias); $n_{g}=\text {10}$ is the number of groups within which validation parameters were compared; and $n_{m}=8$ is the number of unique combinations between methods and use of genomic information.

### Expected pattern in results

The accuracies and biases are expected to differ between populations A, B, and C. For animals in generation 39 of Population A, halfsibs are the closest relatives with phenotypes. For animals in generation 39 of Population B, cousins are the closest relatives with phenotypes. For animals in generation 39 of Population C, own performance is available for all animals. Therefore, we expect that prediction is most accurate in Population C, less accurate in Population A, and least accurate in Population B.

## Results

### Prediction accuracy

Generally, the GT and MF methods were as accurate or more accurate than the SM and NRM methods (Table 2). Use of genomic information always increased the prediction accuracy (Table 2).

For Population A, the methods were equally accurate for prediction of breeding values without genomic information (median: 0.37–0.41, Table 2). When breeding values were predicted with genomic information, the MF and GT methods were the most accurate (median: 0.59–0.65, Table 2). The SM method was generally as accurate as the MF and GT methods (median: 0.58–0.65, Table 2), while the NRM method was always the least accurate (median: 0.56–0.63).

For Population B, the methods were equally accurate for prediction of breeding values without genomic information (median: 0.29–0.35, Table 2). When breeding values were predicted with genomic information, the MF method and the GT method were the most accurate for prediction of any definition of true breeding value (median: 0.50–0.55, Table 2) while the SM and NRM methods were the least accurate (median: 0.48–0.51).

For Population C, the GT and MF methods were the most accurate (median: 0.61–0.63, Table 2). The least accurate methods were the SM method (median: 0.57) and the NRM method (median: 0.48–0.49), respectively (Table 2).

**Table 2:** Median prediction accuracy across replicates

Population $\times$ method	y$_{A}$	y$_{AD}$		
		Purebred	F1	Rotation
Population A (Purebred)				
GT	0.41 (0.09)$^{\mathrm{c}}$	0.37 (0.11)$^{\mathrm{c}}$	0.40 (0.11)$^{\mathrm{c}}$	0.39 (0.12)$^{\mathrm{c}}$
MF	0.41 (0.09)$^{\mathrm{c}}$	0.37 (0.11)$^{\mathrm{c}}$	0.40 (0.11)$^{\mathrm{c}}$	0.39 (0.12)$^{\mathrm{c}}$
NRM	0.39 (0.09)$^{\mathrm{c}}$	0.37 (0.09)$^{\mathrm{c}}$	0.40 (0.11)$^{\mathrm{c}}$	0.37 (0.11)$^{\mathrm{c}}$
SM	0.41 (0.09)$^{\mathrm{c}}$	0.37 (0.10)$^{\mathrm{c}}$	0.41 (0.09)$^{\mathrm{c}}$	0.40 (0.12)$^{\mathrm{c}}$
ssGT	0.65 (0.06)$^{\mathrm{a}}$	0.59 (0.08)$^{\mathrm{a}}$	0.60 (0.08)$^{\mathrm{a}}$	0.60 (0.08)$^{\mathrm{a}}$
ssMF	0.65 (0.06)$^{\mathrm{a}}$	0.59 (0.08)$^{\mathrm{a}}$	0.60 (0.07)$^{\mathrm{a}}$	0.60 (0.08)$^{\mathrm{a}}$
ssNRM	0.63 (0.05)$^{\mathrm{b}}$	0.56 (0.07)$^{\mathrm{b}}$	0.58 (0.06)$^{\mathrm{b}}$	0.58 (0.07)$^{\mathrm{b}}$
ssSM	0.65 (0.04)$^{\mathrm{ab}}$	0.58 (0.08)$^{\mathrm{a}}$	0.60 (0.07)$^{\mathrm{b}}$	0.60 (0.08)$^{\mathrm{a}}$
Population B (Purebred)				
GT	0.34 (0.12)$^{\mathrm{c}}$	0.31 (0.11)$^{\mathrm{c}}$	0.34 (0.12)$^{\mathrm{c}}$	0.34 (0.12)$^{\mathrm{c}}$
MF	0.33 (0.12)$^{\mathrm{c}}$	0.31 (0.12)$^{\mathrm{c}}$	0.34 (0.12)$^{\mathrm{c}}$	0.34 (0.12)$^{\mathrm{c}}$
NRM	0.33 (0.11)$^{\mathrm{c}}$	0.30 (0.11)$^{\mathrm{c}}$	0.31 (0.13)$^{\mathrm{c}}$	0.31 (0.13)$^{\mathrm{c}}$
SM	0.35 (0.11)$^{\mathrm{c}}$	0.29 (0.11)$^{\mathrm{c}}$	0.31 (0.11)$^{\mathrm{c}}$	0.32 (0.11)$^{\mathrm{c}}$
ssGT	0.53 (0.10)$^{\mathrm{a}}$	0.50 (0.08)$^{\mathrm{a}}$	0.54 (0.10)$^{\mathrm{a}}$	0.53 (0.09)$^{\mathrm{a}}$
ssMF	0.55 (0.09)$^{\mathrm{a}}$	0.51 (0.07)$^{\mathrm{a}}$	0.54 (0.08)$^{\mathrm{a}}$	0.54 (0.08)$^{\mathrm{a}}$
ssNRM	0.50 (0.11)$^{\mathrm{b}}$	0.50 (0.09)$^{\mathrm{b}}$	0.50 (0.09)$^{\mathrm{b}}$	0.51 (0.08)$^{\mathrm{b}}$
ssSM	0.51 (0.11)$^{\mathrm{b}}$	0.48 (0.09)$^{\mathrm{b}}$	0.49 (0.08)$^{\mathrm{b}}$	0.50 (0.08)$^{\mathrm{b}}$
Population C (Crossbred)				
GT	0.62 (0.03)$^{\mathrm{b}}$			0.61 (0.03)$^{\mathrm{b}}$
MF	0.62 (0.03)$^{\mathrm{b}}$			0.62 (0.03)$^{\mathrm{b}}$
NRM	0.48 (0.02)$^{\mathrm{f}}$			0.48 (0.02)$^{\mathrm{e}}$
SM	0.57 (0.02)$^{\mathrm{d}}$			0.57 (0.02)$^{\mathrm{d}}$
ssGT	0.63 (0.03)$^{\mathrm{a}}$			0.62 (0.03)$^{\mathrm{a}}$
ssMF	0.63 (0.03)$^{\mathrm{a}}$			0.62 (0.03)$^{\mathrm{a}}$
ssNRM	0.48 (0.02)$^{\mathrm{e}}$			0.49 (0.03)$^{\mathrm{e}}$
ssSM	0.57 (0.02)$^{\mathrm{c}}$			0.57 (0.02)$^{\mathrm{c}}$

Median absolute deviations are in parentheses

Accuracy: Pearson’s correlation between true breeding values and predicted breeding values

Superscripts: Different superscripts denote that medians are significantly different

Superscripts are comparable within combinations of Population and column

ss-prefix: Relationship matrices include genomic information

y$_{A}$: A phenotype with additive genetic effects

y$_{AD}$: A phenotype with both additive and dominant genetic effects

Purebred: True breeding value is for production of purebred animals

F1: True breeding value is for production of F1-animals

Rotation: True breeding value is for mating with rotationally crossbred animals

### Level bias

The level biases were not statistically significantly different from 0 for the phenotype without a dominant genetic term (Table 3). For the phenotype with a dominant genetic term, the level biases were statistically significantly different from 0 for mating an animal with another animal from the same population.

**Table 3:** Median level bias across replicates

Population $\times$ method	y$_{A}$	y$_{AD}$		
		Purebred	F1	Rotation
Population A (Purebred)				
GT	−0.02 (0.08)$^{\mathrm{a}}$	**0.29 (0.09)**$^{\mathrm{a}}$	−0.03 (0.08)$^{\mathrm{a}}$	**0.12 (0.08)**$^{\mathrm{a}}$
MF	−0.02 (0.08)$^{\mathrm{a}}$	**0.28 (0.09)**$^{\mathrm{a}}$	−0.03 (0.08)$^{\mathrm{a}}$	**0.12 (0.08)**$^{\mathrm{a}}$
NRM	−0.01 (0.08)$^{\mathrm{a}}$	**0.29 (0.10)**$^{\mathrm{a}}$	−0.04 (0.09)$^{\mathrm{a}}$	**0.12 (0.09)**$^{\mathrm{a}}$
SM	−0.03 (0.08)$^{\mathrm{a}}$	**0.28 (0.09)**$^{\mathrm{a}}$	−0.03 (0.09)$^{\mathrm{a}}$	**0.11 (0.09)**$^{\mathrm{a}}$
ssGT	−0.01 (0.07)$^{\mathrm{a}}$	**0.28(0.07)**$^{\mathrm{a}}$	−0.01 (0.07)$^{\mathrm{a}}$	**0.13 (0.07)**$^{\mathrm{a}}$
ssMF	−0.01 (0.08)$^{\mathrm{a}}$	**0.28 (0.07)**$^{\mathrm{a}}$	−0.04 (0.07)$^{\mathrm{a}}$	**0.13 (0.08)**$^{\mathrm{a}}$
ssNRM	0.00 (0.08)$^{\mathrm{a}}$	**0.29 (0.08)**$^{\mathrm{a}}$	−0.02 (0.09)$^{\mathrm{a}}$	**0.14 (0.09)**$^{\mathrm{a}}$
ssSM	0.00 (0.07)$^{\mathrm{a}}$	**0.29 (0.08)**$^{\mathrm{a}}$	−0.03 (0.08)$^{\mathrm{a}}$	**0.13(0.08)**$^{\mathrm{a}}$
Population B (Purebred)				
GT	0.00 (0.07)$^{\mathrm{a}}$	**0.28 (0.09)**$^{\mathrm{a}}$	−0.03 (0.08)$^{\mathrm{a}}$	0.04 (0.07)$^{\mathrm{a}}$
MF	0.00 (0.07)$^{\mathrm{a}}$	**0.28 (0.10)**$^{\mathrm{a}}$	−0.02 (0.08)$^{\mathrm{a}}$	0.04 (0.07)$^{\mathrm{a}}$
NRM	0.01 (0.08)$^{\mathrm{a}}$	**0.28 (0.10)**$^{\mathrm{a}}$	0.03 (0.09)$^{\mathrm{a}}$	0.05 (0.08)$^{\mathrm{a}}$
SM	0.01 (0.07)$^{\mathrm{a}}$	**0.29 (0.08)**$^{\mathrm{a}}$	−0.03 (0.08)$^{\mathrm{a}}$	0.04 (0.07)$^{\mathrm{a}}$
ssGT	−0.01 (0.06)$^{\mathrm{a}}$	**0.28 (0.08)**$^{\mathrm{a}}$	−0.03 (0.07)$^{\mathrm{a}}$	0.04 (0.06)$^{\mathrm{a}}$
ssMF	0.01 (0.06)$^{\mathrm{a}}$	**0.28 (0.08)**$^{\mathrm{a}}$	−0.01 (0.07)$^{\mathrm{a}}$	0.05 (0.07)$^{\mathrm{a}}$
ssNRM	0.01 (0.07)$^{\mathrm{a}}$	**0.28 (0.08)**$^{\mathrm{a}}$	−0.02 (0.08)$^{\mathrm{a}}$	0.04 (0.08)$^{\mathrm{a}}$
ssSM	0.01 (0.06)$^{\mathrm{a}}$	**0.28 (0.08)**$^{\mathrm{a}}$	−0.02 (0.07)$^{\mathrm{a}}$	0.04 (0.07)$^{\mathrm{a}}$
Population C (Crossbred)				
GT	0.00 (0.05)$^{\mathrm{a}}$			**0.20 (0.07)**$^{\mathrm{a}}$
MF	0.00 (0.06)$^{\mathrm{a}}$			**0.20 (0.07)**$^{\mathrm{a}}$
NRM	0.00 (0.06)$^{\mathrm{a}}$			**0.19 (0.07)**$^{\mathrm{a}}$
SM	0.00 (0.06)$^{\mathrm{a}}$			**0.20 (0.06)**$^{\mathrm{a}}$
ssGT	0.00 (0.06)$^{\mathrm{a}}$			**0.20 (0.07)**$^{\mathrm{a}}$
ssMF	0.00 (0.06)$^{\mathrm{a}}$			**0.20 (0.07)**$^{\mathrm{a}}$
ssNRM	0.00 (0.06)$^{\mathrm{a}}$			**0.21 (0.06)**$^{\mathrm{a}}$
ssSM	0.00 (0.06)$^{\mathrm{a}}$			**0.20 (0.06)**$^{\mathrm{a}}$

Median absolute deviations from medians are in parentheses

Level Bias: Difference between change in predicted and true breeding values relative to in the base population

Bold: Medians in bold differ significantly from zero

Superscripts: Different superscripts denote that medians are significantly different

Superscripts are comparable within combinations of Population and column

ss-prefix: Relationship matrices include genomic information

y$_{A}$: A phenotype with additive genetic effects

y$_{AD}$: A phenotype with both additive and dominant genetic effects

Purebred: True breeding value is for production of purebred animals

F1: True breeding value is for production of F1-animals

Rotation: True breeding value is for mating with rotationally crossbred animals

### Dispersion bias

In general, the dispersion biases for the GT, MF, and SM methods were not statistically significantly different from 1 (Table 4). 

For Population A, the dispersion biases for the GT and MF methods were not statistically significantly different from 1 for the phenotype without a dominant genetic term (median: 0.94–1.01). The dispersion biases for SM method were not statistically significantly different from 1 when breeding values were predicted without genomic information or the phenotype was without a dominant genetic term (median: 0.78–0.94). The dispersion biases for the NRM method were always statistically significantly different from 1.

For Population B, the dispersion biases for the GT and MF methods were not statistically significantly different from 1 (median: 0.91–1.12). The dispersion biases for the SM method were only statistically significantly different from 1 when breeding values were predicted with genomic information and the phenotype did not include a dominant genetic term (median: 1.20). The dispersion biases for the NRM method was statistically significantly different from 1 in almost all cases (median: 0.67–0.82).

For Population C, the dispersion biases for the GT and MF methods were not statistically significantly different from 1 when the phenotype did not include a dominant genetic term (median: 0.99–1.02). The dispersion biases for the SM and NRM methods were always statistically significantly different from 1.

**Table 4:** Median dispersion bias across replicates

Population $\times$ method	y$_{A}$	y$_{AD}$		
		Purebred	F1	Rotation
Population A (Purebred)				
GT	0.98 (0.22)$^{\mathrm{ab}}$	0.85 (0.22)$^{\mathrm{ab}}$	0.89 (0.28)$^{\mathrm{ab}}$	0.86 (0.27)$^{\mathrm{ab}}$
MF	0.96 (0.22)$^{\mathrm{ab}}$	0.84 (0.20)$^{\mathrm{b}}$	0.88 (0.24)$^{\mathrm{ab}}$	0.85 (0.24)$^{\mathrm{ab}}$
NRM	**0.74 (0.17)**$^{\mathrm{c}}$	**0.62 (0.17)**$^{\mathrm{c}}$	**0.65 (0.15)**$^{\mathrm{c}}$	**0.65 (0.18)**$^{\mathrm{c}}$
SM	0.94 (0.24)$^{\mathrm{ab}}$	0.78 (0.25)$^{\mathrm{b}}$	0.84 (0.26)$^{\mathrm{b}}$	0.79 (0.25)$^{\mathrm{b}}$
ssGT	1.01 (0.14)$^{\mathrm{a}}$	**0.90 (0.15)**$^{\mathrm{a}}$	0.96 (0.14)$^{\mathrm{a}}$	0.95 (0.14)$^{\mathrm{a}}$
ssMF	1.00 (0.11)$^{\mathrm{ab}}$	**0.88 (0.12)**$^{\mathrm{ab}}$	0.93 (0.12)$^{\mathrm{ab}}$	**0.90 (0.11)**$^{\mathrm{ab}}$
ssNRM	**0.76 (0.10)**$^{\mathrm{c}}$	**0.67 (0.11)**$^{\mathrm{c}}$	**0.69 (0.10)**$^{\mathrm{c}}$	**0.66 (0.10)**$^{\mathrm{c}}$
ssSM	0.94 (0.15)$^{\mathrm{b}}$	**0.84 (0.18)**$^{\mathrm{b}}$	**0.86 (0.15)**$^{\mathrm{b}}$	**0.84 (0.17)**$^{\mathrm{b}}$
Population B (Purebred)				
GT	1.12 (0.40)$^{\mathrm{bc}}$	1.02 (0.44)$^{\mathrm{a}}$	1.01 (0.43)$^{\mathrm{a}}$	1.01 (0.41)$^{\mathrm{a}}$
MF	1.10 (0.37)$^{\mathrm{bc}}$	0.98 (0.39)$^{\mathrm{a}}$	1.02 (0.42)$^{\mathrm{a}}$	0.96 (0.44)$^{\mathrm{a}}$
NRM	0.82 (0.30)$^{\mathrm{d}}$	**0.72 (0.33)**$^{\mathrm{b}}$	**0.74 (0.34)**$^{\mathrm{b}}$	**0.73 (0.33)**$^{\mathrm{b}}$
SM	1.26 (0.57)$^{\mathrm{a}}$	0.94 (0.47)$^{\mathrm{a}}$	1.15 (0.51)$^{\mathrm{a}}$	1.13 (0.50)$^{\mathrm{a}}$
ssGT	1.09 (0.16)$^{\mathrm{c}}$	0.98 (0.20)$^{\mathrm{a}}$	0.99 (0.17)$^{\mathrm{a}}$	0.96 (0.16)$^{\mathrm{a}}$
ssMF	1.04 (0.17)$^{\mathrm{c}}$	0.91 (0.19)$^{\mathrm{a}}$	0.93 (0.16)$^{\mathrm{a}}$	0.93 (0.15)$^{\mathrm{a}}$
ssNRM	**0.80 (0.16)**$^{\mathrm{d}}$	**0.67 (0.15)**$^{\mathrm{b}}$	**0.68 (0.14)**$^{\mathrm{b}}$	**0.67 (0.12)**$^{\mathrm{b}}$
ssSM	**1.20 (0.30)**$^{\mathrm{ab}}$	1.02 (0.27)$^{\mathrm{a}}$	0.98 (0.23)$^{\mathrm{a}}$	0.97 (0.22)$^{\mathrm{a}}$
Population C (Crossbred)				
GT	1.00 (0.06)$^{\mathrm{a}}$			**0.92 (0.06)**$^{\mathrm{a}}$
MF	1.01 (0.06)$^{\mathrm{a}}$			**0.93 (0.05)**$^{\mathrm{a}}$
NRM	**0.40 (0.04)**$^{\mathrm{e}}$			**0.34 (0.03)**$^{\mathrm{e}}$
SM	**0.54 (0.05)**$^{\mathrm{c}}$			**0.46 (0.04)**$^{\mathrm{c}}$
ssGT	0.99 (0.07)$^{\mathrm{a}}$			**0.92 (0.06)**$^{\mathrm{a}}$
ssMF	1.00 (0.07)$^{\mathrm{a}}$			**0.93 (0.05)**$^{\mathrm{a}}$
ssNRM	**0.41 (0.04)**$^{\mathrm{d}}$			**0.34 (0.03)**$^{\mathrm{d}}$
ssSM	**0.55 (0.05)**$^{\mathrm{b}}$			**0.46 (0.04)**$^{\mathrm{b}}$

Median absolute deviations from medians are in parentheses

Dispersion Bias: Linear regression coefficient of true breeding values onto predicted breeding values

Bold: Medians in bold differ significantly from zero

Superscripts: Different superscripts denote that medians are significantly different

Superscripts are comparable within combinations of Population and column

ss-prefix: Relationship matrices include genomic information

y$_{A}$: A phenotype with additive genetic effects

y$_{AD}$: A phenotype with both additive and dominant genetic effects

Purebred: True breeding value is for production of purebred animals

F1: True breeding value is for production of F1-animals

Rotation: True breeding value is for mating with rotationally crossbred animals

## Discussion

As hypothesized, the GT and MF methods were generally the most accurate and least biased methods for prediction of breeding values with phenotypes from rotationally crossbred animals. The SM method was almost as accurate as the GT and MF methods but was also more biased. The NRM method was the least accurate and most biased of the methods.

### The GT and MF methods

We found that the GT and MF methods performed similarly for prediction of breeding values with phenotypes from rotationally crossbred animals. This is in accordance with the fact that the MF method, in theory, can account for both breed-specific terms and segregation terms from GT method [14]. More specifically, the GT and MF methods are equivalent when the tranformations of Eq. 22 yield the estimated variance components from the GT method. However, this relies on the accurate estimation of the metafounder relationships which has some degree of estimation error. Fortunately for the MF method, it is the relative sizes of $\gamma _A$, $\gamma _B$, and $\gamma _{AB}$ which determine the relative sizes of the partial additive genetic parameters, $\sigma ^2_{A_A}$, $\sigma ^2_{A_B}$, and $\sigma ^2_{A_{AB}}$ (Eq. 22). As long as Eq. 22 holds true, changes to the metafounder relationships are accounted for through changes to the estimated additive genetic variance in the ancestral population, $\sigma ^2_{MF}$.

One major advantage of the MF method is that genomic information can readily be included in the additive relationship matrix with metafounders using the single-step procedure [14], regardless of the genetic composition of the animals in the relationship matrix. On the contrary, the single-step procedure has only been developed for the partial relationship matrices for breed-specific terms from the GT method; i.e., a combined partial relationship matrix for both genotyped and non-genotyped animals for segregation terms has not been developed [6]. This may make the MF method more appropriate than the GT method when rotationally crossbred animals are genotyped.

Based on this study, it is not possible to conclude whether the GT or MF method is better for the analysis of these specific populations.

#### The SM method

This method was generally as accurate and unbiased as the GT and MF methods for prediction in purebred animals but less accurate and more biased for prediction in rotationally crossbred animals (Tables 2 and 4). The inaccuracy and bias of the SM method may be caused by its inability to properly separate the phenotype into its components (Table 1).

The SM method is only an approximation to the GT method and discreprancies between the two are expected. For example, for the GT method and disregarding inbreeding, the covariance between siblings depends on the diagonal elements of their shared parents (Eq. 7). Meanwhile, for the SM method, the covariance between siblings depends on the product between their own regression covariates for partial additive genetic effects (Eqs. 11, 12, 13). Consequently, the SM method is a better approximation to the GT method between animals where the weighted average of diagonal and off-diagonal elements of common ancestors is equal to the product between the animals’ regression covariates for partial additive genetic effects and their additive genetic covariance according to the NRM method.

In a rotational crossbreeding system, breed proportions differ across generations. Consequently, the weighted average of diagonal and off-diagonal elements of common ancestors can differ from the product between the animals’ regression covariates for partial additive genetic effects and their additive genetic covariance according to the NRM method. For example, in this study and disregarding inbreeding, the covariance of partial additive breed-specific effects from Population A between full sibs *i* and *j* from generation 34 and Population C was not the same for the GT and SM methods:

30 $$\begin{aligned} \begin{aligned} a_{ij}^{SM} =&\sqrt{f^A_if^A_j}\frac{1}{4}\left[ a_{s}^{NRM:A} + a_{d}^{NRM:A}\right] = \sqrt{\frac{1}{4}\frac{1}{4}}\frac{1}{4}\left[ 0 + 1\right] = \frac{1}{16}, \\ a_{ij}^{GT} =&\frac{1}{4}\left[ f^A_{s} + f^A_{d}\right] = \frac{1}{4}\left[ 0 + \frac{1}{2}\right] = \frac{1}{8}, \end{aligned} \end{aligned}$$

where subscripts *i*, *j*, *s*, and *d* denote animals; $f^A$ is the breed proportion from Population A; and superscript *NRM:A* denotes that the covariance was calculated with the NRM method and the pedigree tracing breed-specific genetic effects from Population A. Meanwhile, the covariance for partial additive breed-specific effects from Population B between the same animals was the same for the GT and SM methods:

31 $$\begin{aligned} \begin{aligned} a_{ij}^{SM} =&\sqrt{f^B_if^B_j}\frac{1}{4}\left[ a_{s}^{NRM:B} + a_{d}^{NRM:B}\right] = \sqrt{\frac{3}{4}\frac{3}{4}}\frac{1}{4}\left[ 1 + 1\right] = \frac{3}{8}, \\ a_{ij}^{GT} =&\frac{1}{4}\left[ f^B_{s} + f^B_{d}\right] = \frac{1}{4}\left[ 1 + \frac{1}{2}\right] = \frac{3}{8}, \end{aligned} \end{aligned}$$

where superscript *NRM:B* denotes that the covariance was calculated with the NRM method and the pedigree that traces breed-specific genetic effects from Population B; $f^B$ is the breed proportion from Population B; and the other terms are as for Eq. 30.

It is simple to see that the GT and SM methods do not always produce identical relationships. However, it is challenging to explain how discreprancies between the GT and SM methods across the three partial additive relationship matrices affect the partitioning of random effects. Nevertheless, according to our study, the SM method seems to be a good approximation of the GT method when the aim is to predict breeding values in purebred animals.

#### The NRM method

This method has the most inaccurate assumptions for additive genetic effects among the methods investigated. In rotationally crossbred animals between divergent purebred populations, the model does not fit the data if the partial additive genetic variances due to breed-specific effects are not proportional to breed proportions [10], and the segregation variance is not modelled [4]. Therefore, it was expected that this method was the least accurate and most biased among those investigated (Tables 2, 3, 4).

The NRM method is a common approach for multi-breed analyses. The main argument for the NRM method is that it is commonly implemented into softwares for genetic evaluations. However, we argue that the GT, MF, and SM methods either are accessible or can easily become accessible. Currently, the GT or MF methods may not be implemented in softwares for genetic evaluations, but both random regression and the NRM method are. The combination of random regression and the NRM method enables the use of the SM method which, in this study, was more accurate and less biased than the NRM method (Tables 2, 3, 4). In the future, the GT and MF methods should become accessible through their implementation into commonly used softwares for genetic evaluations. The implementation of both the GT and MF methods is simple as the algorithms for directly computing their inverse covariance matrices are very similar to the algorithm for the NRM method [9, 14]. Consequently, the time required for implementing the GT and MF methods should be greatly reduced as a large proportion of program code from the NRM method can be reused. All things considered, we do not recommend the NRM method for genetic analyses with phenotypes of rotationally crossbred animals, because its alternatives are more accurate, less biased, and easily accessible.

#### Simulation design

Results from simulation studies are most relevant when the simulated populations are representative of real populations. Populations can be described with several parameters, however, the divergence between the populations is a key argument for the relevance of multibreed relationship matrices [4]. The magnitude of divergence between two populations can be represented by the ratio between the segregation variance and the additive genetic variance in F2 animals: $\sigma ^2_{AB}/\left( \frac{1}{2}\sigma ^2_A + \frac{1}{2}\sigma _B^2 + \sigma ^2_{AB}\right)$; which, in turn, can be calculated using the metafounder relationships (Eq. 22). Using this measure, the average magnitude of divergence between Populations A and B is 15% based on the metafounder relationships (Table 1). Meanwhile, this measure for the magnitude of divergence is 16% between DanBred Landrace and DanBred Yorkshire pigs [32], 15% between Hereford and Zebu cattle [33], and on average 11% (min: 3%, max: 25%) between subpopulations of Manech Tête Rousse sheep [34]. Therefore, the magnitude of divergence between populations A and B is representative of the divergence between real populations.

It would have been reasonable to compare the methods with a different simulation design, which would most likely give a different result. However, the purebred populations need to have diverged from each other; otherwise segregation effects would be small. We ensured that the purebred populations had diverged by simulating separate population bottlenecks in the two populations, and not a shared population bottleneck; by only sampling 50 animals (0.2% of the historical population) when founding the purebred populations; by keeping the effective population sizes small in the purebred populations ($N_e\approx 50$ animals); and by isolating the purebred populations for 32 generations prior to the pedigreed generations. In a scenario where the purebred populations had only slightly diverged from each other, segregation effects would be small and the additive genetic variances would be the same in the purebred populations. This would diminish the argument for partial additive relationship matrices for the breed-specific terms and the segregation term. In other words, it would be better to regard the two purebred populations as one purebred population. A simulation design with less diverged purebred populations would most likely yield the same ranking of the methods but with less absolute differences between their prediction accuracies.

In this study, only genetic drift caused changes in allele frequencies. In practice, allele frequencies are also affected by selection. Simulating selection would most likely also change the results. However, we have no reason to believe that selection would change the ranking between the methods, because all the methods theoretically can account for selection, and because their mechanism for doing so is the same [8, 9, 12, 14].

#### Genotypes from crossbred animals

It is simpler to incorporate genomic information from crossbred animals into some methods than into others. For the MF, SM, and NRM methods, genomic information on crossbreds can be incorporated as for purebred animals. For the GT method, it becomes necessary to trace the breed of origin of alleles to construct genomic relationship matrices for breed-specific terms [13]. Furthermore, to our knowledge, it is not known how genomic information should be incorporated into partial relationship matrices for segregation terms. Although it is simple to incorporate genomic information for the MF, SM, and NRM methods, it is not known whether the resulting relationship matrices correctly represent the additive genetic covariance between animals. In particular, this is the case for the SM method and our application of the NRM method, as they are approximations. Although relevant, it was outside the scope of this study to compare the methods in a scenario with genomic information from crossbred animals.

#### Synthetic breeds

This study was on genetic analyses with rotationally crossbred animals, but our results may also apply to other genetic analyses of mixed populations. For example, some breeding companies create synthetic breeds. In practice, synthetic breeds are crossbred populations and they are subject to the same mechanisms as other crossbred populations. The only difference between a rotationally crossbred population and a synthetic breed is that sires are not necessarily purebred for synthetic breeds. Similar to the rotationally crossbred populations, the complex distributions of genetic effects may complicate accurate and unbiased prediction of breeding values in synthetic breeds. Our results may assist with the choice of method for the relationship matrix used in genetic analysis of synthetic breeds.

#### Solving BLUP equation systems

The choice between methods may also be impacted by their computational requirements. For all the relationship matrices that were studied here, the inverse can be directly computed [8, 9, 14]. However, the resulting equation systems differ in dimensions and sparseness. Using the GT, SM, or NRM method results in a larger equation system than with the MF method; especially with large numbers of breeds and crossbred animals. Meanwhile, the MF method contains more non-zero elements than the other methods; and using the MF method with the single-step procedure may require the inversion of one large genomic relationship matrix rather than the inversion of smaller genomic relationship matrices as with the other methods. Comparison of computational demands between the methods was outside the scope of this study but it could be relevant when computer hardware is a limiting factor.

## Conclusion

In the scenarios that we investigated, models using the additive relationship matrix with metafounders [14] or the partial relationship matrices by García-Cortés and Toro [9] were generally more accurate and less biased than those using the partial relationship matrices by Strandén and Mäntysaari [12] or the usual numerator relationship matrix [8].

## Data Availability

The datasets used and analysed during the current study are available from the corresponding author on reasonable request.

## Appendix

### Appendix 1: Multibreed relationship matrices with a small example pedigree

The differences between the NRM, GT, and SM methods are easier to understand through examples. This example is based on a pedigree with both purebred animals, F1 animals, F2 animals, and a F2-backcross animal (Table 5). We use the GT method as reference because it is theoretically correct.

**Table 5:** Example pedigree

Id	Sire	Dam	Breed
1	0	0	A
2	0	0	B
3	0	0	A
4	0	0	B
5	1	2	–
6	3	4	–
7	5	6	–
8	5	6	–
9	1	7	–

The methods yield different additive relationship matrices for the term from breed A (Tables 6, 7, 8). The NRM method calculates the correct relationships for purebred animals; but is erroneous after it encounters crossbred animals. The diagonal elements for crossbred animals are not scaled according to their breed proportions, and this error affects both diagonal and off-diagonal elements for descendants of the crossbred animals (Tables 6 and 7). The SM method yields the same diagonal elements as the GT method in the absence of inbreeding (Table 8). The off-diagonal elements between F1 and F2 crossbred animals are also correct. The off-diagonal elements between purebred animals and crossbred animals are erroneous, and so is the off-diagonal element for the F2-backcross (animal 9; Table 8).

**Table 6:** Breed-specific relationship matrix for the GT-method with the sample pedigree

$\mathbf{A }^{GT}_{A}$	1	3	5	6	7	8	9
1	1.00						
3		1.00					
5	0.50		0.50				
6		0.50		0.50			
7	0.25	0.25	0.25	0.25	0.50		
8	0.25	0.25	0.25	0.25	0.25	0.50	
9	0.63	0.13	0.38	0.13	0.38	0.25	0.88

Upper triangle and zeroes are omitted

**Table 7:** Breed-specific relationship matrix for the NRM-method with the sample pedigree

$\mathbf{A }^{NRM}_{A}$	1	3	5	6	7	8	9
1	1.00						
3		1.00					
5	0.50		1.00				
6		0.50		1.00			
7	0.25	0.25	0.50	0.50	1.00		
8	0.25	0.25	0.50	0.50	0.50	1.00	
9	0.63	0.13	0.50	0.25	0.63	0.38	1.13

Upper triangle and zeroes are omitted

**Table 8:** Breed-specific relationship matrix for the SM-method with the sample pedigree

$\mathbf{A }^{SM}_{A}$	1	3	5	6	7	8	9
1	1.00						
3		1.00					
5	0.35		0.50				
6		0.35		0.50			
7	0.18	0.18	0.25	0.25	0.50		
8	0.18	0.18	0.25	0.25	0.25	0.50	
9	0.54	0.11	0.31	0.15	0.38	0.23	0.84

Upper triangle and zeroes are omitted

The methods also yield different partial additive relationship matrices for the segregation term (Tables 9 and 10). The SM method calculates non-zero off-diagonal elements for related animals where the off-diagonal element is zero for the GT method. Furthermore, the off-diagonal elements between animals 7 and 9 are erroneous as is the diagonal element for animal 9.

**Table 9:** Relationship matrix for the segregation term with sample pedigree and the GT-method

$\mathbf{A }_{AB}^{GT}$	7	8	9
7	1.00		
8		1.00	
9	0.50		0.50

Upper triangle and zeroes are omitted

**Table 10:** Relationship matrix for the segregation term with sample pedigree and the SM-method

$\mathbf{A }_{AB}^{SM}$	7	8	9
7	1.00		
8	0.50	1.00	
9	0.44	0.27	0.56

Upper triangle and zeroes are omitted

The relationship matrix from the MF method is not directly comparable to those from the other methods (Table 11) although it is theoretically equal to the GT method [14].

**Table 11:** Relationship matrix with sample pedigree and the MF-method

$\mathbf{A }^{MF}$	1	2	3	4	5	6	7	8	9
1	1.33								
2	0.10	1.25							
3	0.66	0.10	1.33						
4	0.10	0.50	0.10	1.25					
5	0.72	0.68	0.38	0.30	1.05				
6	0.38	0.30	0.72	0.68	0.34	1.05			
7	0.55	0.49	0.55	0.49	0.70	0.70	1.17		
8	0.55	0.49	0.55	0.49	0.70	0.70	0.70	1.17	
9	0.94	0.29	0.60	0.29	0.71	0.54	0.86	0.62	1.27

Upper triangle is omitted. The matrix was calculated with: $\gamma _{A}=0.66$, $\gamma _{AB}=0.1$, and $\gamma _{B}=0.50$
